# Raspberry Pomace as a Good Additive to Apple Freeze-Dried Fruit Bars: Biological Properties and Sensory Evaluation

**DOI:** 10.3390/molecules29235690

**Published:** 2024-12-01

**Authors:** Urszula Szymanowska, Monika Karaś, Anna Jakubczyk, Janusz Kocki, Rafał Szymanowski, Ireneusz Tomasz Kapusta

**Affiliations:** 1 Department of Biochemistry and Food Chemistry, University of Life Sciences, 8 Skromna Str., 20-704 Lublin, Poland; monika.karas@up.lublin.pl (M.K.); anna.jakubczyk@up.lublin.pl (A.J.); 2 Department of Clinical Genetics, Medical University of Lublin, 11 Radziwiłłowska Str., 20-080 Lublin, Poland; janusz.kocki@umlub.pl (J.K.); rafal.szymanowski@umlub.pl (R.S.); 3 Department Food Technology and Human Nutrition, Institute of Food Technology, College of Natural Science, University of Rzeszów, 4 Zelwerowicza Str., 35-601 Rzeszów, Poland; ikapusta@ur.edu.pl

**Keywords:** freeze-dried apple/raspberry bars, pomace, antioxidant, anti-inflammatory, antihypertensive, antiproliferative, potential bioaccessibility

## Abstract

This study investigated the impact of adding raspberry pomace to the phenolic content and biological properties of freeze-dried apple/raspberry bars. The bars were prepared by replacing apple puree with raspberry pomace (5–50%), and their phenolic compounds were assessed using ethanol and buffer extracts. This work also explored the potential bioaccessibility of phenolic compounds in enriched bars through a simulated digestion process (digest). Antioxidant, anti-inflammatory (LOX, COX-2 inhibition), antihypertensive (ACE inhibition), and antiproliferative effects on AGS and HT-29 cancer cells were evaluated. The total polyphenol content was highest in the all bar variants post-digestion. The highest—904.26 ± 23.5 mg/100 g—was determined for the B50 sample In the enriched bars, the concentration of chlorogenic acid decreased from 6.99 ± 1.08 mg/L for BP5 to 2.75 ± 0.32 mg/L for BP50, but the ellagic acid concentration increased from 1.46 ± 0.02 mg/L for BP5 to 12.73 ± 0.09 mg/L for BP50. Among the tested extracts, the highest antioxidant and LOX, COX-2 inhibiting activity was determined for digest. The ability to neutralize free radicals increased with raspberry pomace addition from 3.63 ± 0.26 mM TE/100 g for BC to 5.58 ± 0.22 mM TE/100 g for the BP50 sample. ACE inhibition was quite similar for ethanolic and digest extracts, but much weaker for buffer extracts. The lowest EC50 value was 1.04 ± 0.03 mg/mL for the BP30 ethanolic sample. Analyzed extracts showed antiproliferative activity against both tested cell lines. The EC50 values for HT-29 cancer cells decreased from 0.354 ± 0.031 mg/mL for BC to 0.026 ± 0.006 mg/mL for the BP50 digest sample. It can be assumed that the BP30 bar best met the assumed criteria, and is optimal for both sensory quality (receiving an average score of 4.45) and health benefits.

## 1. Introduction

Lifestyle diseases have become a very serious problem for all developing and developed countries, including Poland. This applies in particular to obesity, hypertension, ischemic heart disease and chronic peptic ulcer disease, and allergic diseases, along with diabetes (mainly type 2). Chronic inflammation often coexists with these diseases. Polyphenols, especially from the group of flavonoids, contained in many vegetables and fruits, find application in their prevention and supporting treatment [1].

Apples are a very popular fruit eaten fresh. They are easy to store, and are also an excellent base for the production of healthy snacks, especially since they contain significant amounts of fiber, vitamins, and phenolic compounds [2]. Raspberries, on the other hand, are very perishable fruits, and are often processed into juices. The remaining pomace poses a disposal problem due to its susceptibility to microbial contamination. On the other hand, they are very rich in biologically active compounds, especially ellagitannins and flavonoids, including anthocyanins [3]. Raspberry pomace contains significant amounts of seeds, which, when eaten whole, pass through the digestive tract unchanged, constituting an additional source of dietary fiber [4].

The diet of many modern people is based on highly processed products, which are easy to prepare but are devoid of many biologically active substances. With the growing consumer interest in functional foods in recent years, there is a demand for easy-to-prepare and even ready-to-use functional snacks [5]. The condition for their production on an industrial scale is the development of recipes on a laboratory scale. An additional advantage in the production of such snacks may be the use of the freeze-drying technique, as low temperature prevents the degradation of labile compounds, especially anthocyanins. Freezing, primary drying, and secondary drying are the main steps required to obtain maximum product quality in the freeze-drying process. The key parameters of the technique are temperature and time, which affect the content of bioactive compounds and the taste properties of the products. The freezing and primary drying processes depend on the size and quantity of ice crystals, and especially on the time of their application. Large and small ice crystals obtained in the slow cooling process allow for rapid sublimation and shorten the drying time. On the other hand, the degradation of the material cells, which results from the formation of large ice crystals, leads to an increase in the amount of some bioactive components, which are less in fresh food compared with freeze-dried material. After freezing, the ice crystals are sublimated directly into the gas phase at a low pressure and temperature, without passing through the liquid phase, which protects the valuable components from degradation. In the last step—secondary drying—the temperature is increased and the pressure is reduced for efficient drying. Higher temperatures are not harmful to food at this stage because there are no more ice crystals and therefore no liquid phase is formed [6]. In addition, the risk of contamination of the material with pathogenic bacteria is reduced, and this process is conducive to the long-term storage of the obtained products [7].

The biological activity of polyphenols depends mainly on the changes in the human digestive system after consumption of products containing these compounds, along with the structure of the resulting metabolites. Phenolic acids and flavonoids are characterized by the greatest activity, especially antioxidant activity, but it should be noted that it is related to the structure of molecules, in particular the number and distribution of hydroxyl groups [8]. The biological activity of phenolic compounds also includes properties resulting indirectly from anti-free radical activity, i.e., anti-inflammatory, antimicrobial, or anticancer properties [9,10,11]. Polyphenols exhibit antioxidant activity, including neutralizing free radicals or inhibiting the activity of enzymes involved in their formation, chelating metal ions, or preventing lipid peroxidation. The greatest effectiveness in carrying out these reactions is demonstrated by flavonoids, which bind hydroxyl radicals, which pose the greatest danger to the human body [12]. The anti-inflammatory activity of natural phenolic compounds may be explained by several mechanisms, including neutralizing free radicals, regulating the activity of cells associated with inflammation, influencing the activity of enzymes involved in the metabolism of arachidonic acid (phospholipase A2, lipoxygenase, cyclooxygenase) and inducible nitric oxide synthase (iNOS), regulating the production of pro-inflammatory compounds (transcription factors, e.g., NF-κB, pro-inflammatory cytokines), or modulating the expression of pro-inflammatory genes [13]. The anticancer effects of phenolic compounds include, among others, protection against excess free radicals and DNA damage, promotion of apoptosis, or inhibition of growth and proliferation of cancer cells. Great hopes in the chemoprevention of colon cancer are associated with the biological activity of anthocyanins. The relatively low level of anthocyanin penetration into the bloodstream in the small intestine may be a significant factor limiting the development of this type of cancer. It is significant that these compounds have a selective proapoptotic effect on cancer cells, but do not affect the growth and division of normal cells [14].

In recent years, research has been conducted on the fortification of products with pomace from various berries. However, the focus was mainly on technological and sensory parameters or only on antioxidant properties. There is little research on the broadly understood pro-health properties of the final products obtained [15,16,17,18]. Thus, the aim of this study was to investigate the effect of the addition of raspberry pomace on the content of phenolic compounds and the broadly understood biological/pro-health activity of freeze-dried apple/raspberry bars. This goal was achieved by determining the content of phenolic compounds, antioxidant activity, the impact on enzymes involved in the pathogenesis of inflammation (LOX (lipoxygenase) and COX-2 (cyclooxygenase-2)), and potential antihypertensive activity (inhibition of ACE (angiotensin-converting enzyme)) of ethanolic and buffer extracts obtained from bars, in which apple mousse used as a bar base was partially replaced with raspberry pomace in the amounts of 5%, 10%, 20%, 30%, and 50%. This work also attempted to determine the potential bioaccessibility of phenolic compounds from bars enriched with raspberry pomace by using a simulated digestion process. Additionally, we checked the impact of obtained extracts on the viability of two digestive tract cancer cell lines.

## 2. Results

### 2.1. Phenolics Analysis

**Figure 1:**
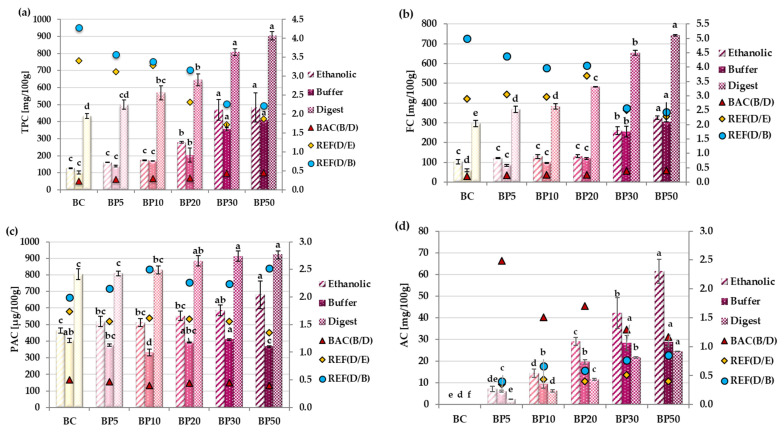
Phenolic compound content in the ethanolic and buffer extract; extract after in vitro digestion of analyzed bars. (**a**) Total phenolic content; (**b**) flavonoid content; (**c**) phenolic acid content; (**d**) anthocyanin content. BC—control (only apple) bars; BP5–BP50—bars with raspberry pomace addition (from 5 to 50% apple mousse substitution, respectively). REF—relative extractability factor, BAC—bioaccessibility index. All values are mean ± standard deviation for triplicate experiments. Means (±SD) followed by different letters are significantly different (n = 9; *p* ≤ 0.05) within the same type of extract.

The estimation of total phenolic, flavonoid, phenolic acid, and anthocyanin content in extracts from apple/raspberry bars is presented in Figure 1. The content of total phenolic compounds in the tested extracts of freeze-dried apple bars with the addition of raspberry pomace expressed as gallic acid equivalent increased with increasing pomace addition.

The use of different solvents for extraction can affect the content of phenolic compounds. Extraction with organic solvents such as ethanol is the most commonly used method, but it does not reflect the situation in the body. In turn, buffer extraction is mainly used to isolate hydrophilic compounds, which can lead to an underestimation of the amount of phenolic compounds, but can simulate the situation before digestion. The most interesting seems to be the determination of the content of polyphenols and their antioxidant potential of samples subjected to simulated digestion, which allows the determination of the content of potentially bioavailable compounds. The lowest polyphenol content was recorded in buffer extracts and ranged from 101.40 mg/100 g in the BC sample without the addition of raspberry pomace to 407.25 mg/100 g in the BP50 variant with a 50% addition of pomace. Some phenolic compounds were difficult to extract from hydrophilic conditions (buffer extract); only water-soluble compounds passed into the solution. A slightly higher content of phenolic compounds was recorded in the ethanol extracts. Statistically significant differences from the control were noted for samples BP20–BP50. The determined content of total polyphenols was the highest in all bar variants after in vitro digestion. The increase in the content of total phenols in digest compared with ethanolic and buffer extracts proved the release of these compounds from the food matrix. Changes in pH and the activity of digestive enzymes may cause the release of phenolic compounds bonded to carbohydrates, increasing their potential bioaccessibility. The relative extractability factor (REF) values, determined for the extracts after digestion from ethanolic and buffer, were higher than 1, and REF values decreased with the increase in the addition of raspberry pomace to the bars, probably due to the higher share of unstable anthocyanins in the enriched bars. However, this did not change the fact that, after in vitro digestion, the concentration of phenolic compounds was about 2 to 4 times higher than before. BAC values below 1 are intended to indicate the high efficiency of digestion in releasing phenolic compounds from the food matrix. It is worth noting that the BAC values increased with the addition of raspberry pomace ranging from 0.23 to 0.45, which proved the positive effect of pH changes and enzyme action on the potential bioaccessibility of phenolics in comparison with the situation before digestion (buffer extract).

A very similar trend was also observed concerning total flavonoid content (Figure 1b). In all types of analyzed extracts, their content increased with the increase in the share of raspberry pomace. However, statistically significant differences between control bars in ethanolic and buffer extracts were found only for 30% and 50% of the addition. In digest, these statistical differences were found even for the BP5 sample relative to the control. The relative bioavailability factors for all analyzed samples were above 2; hence, the flavonoids from the freeze-dried bars were accessible in vitro. The BAC index increased from 0.2 to 0.41 with the addition of the pomace.

The content of phenolic acids increased in the ethanolic extracts with the addition of pomace (Figure 1c). This was confirmed by qualitative and quantitative analysis using UPLC-PDA-MS/MS (Table 1). The main phenolic acid in raspberries is ellagic acid, the concentration of which increased with the addition of pomace to the bars. Chlorogenic acids and coumaric acid derivatives predominate in apples. Their content decreased successively when part of the apple mass was replaced with raspberry pomace. In the buffer extracts (Figure 1c), the content of phenolic acids was much lower than in the ethanolic extracts. This may be due to the low solubility of ellagic acid in aqueous solutions. During in vitro digestion, the amount of phenolic acids increased statistically significantly only in sample BP20 (Figure 1c). pH changes and enzymatic hydrolysis probably released significant amounts of ellagic acid from ellagitannins. However, its previously mentioned low solubility in water means that it did not pass into the “after digestion” solution used for determinations. Anthocyanins, next to ellagitannins, are the main group of polyphenols in raspberry fruits and the pomace obtained from them. The presence of anthocyanins was not detected in the control bars because they were not present in the pulp of apples of this variety. Their content increased with the increase in the addition of raspberry pomace (Figure 1d). The highest concentrations were determined for ethanolic extracts. The content of anthocyanins in the buffer extracts was lower than in the ethanolic extracts, and the differences increased with the increase in the addition of pomace. Although anthocyanins are highly soluble in water, alcohol is a better extractant. The lowest anthocyanin content was determined in digest. This group of phenolic compounds is sensitive to the changing of pH, as occurs during digestion. The extracts obtained after gastrointestinal digestion are alkaline, which results in the transformation of the anthocyanins into a blue anionic quinoid base, the presence of which is also masked by colored bile salts. Without purifying the extract on Sep-Pak C-18 cartridges, it was impossible to determine the anthocyanin content using the standard pH differential method. This may lead to an underestimation of their content. Relative extractability factors (REFs) increased with the addition of raspberry pomace, while the bioaccessibility index (BAC) decreased significantly (Figure 1d).

The identification of polyphenolic compounds in the control bars and the bars fortified with raspberry pomace is presented in Table 1. The identification of polyphenolic compounds in freeze-dried apple bars enriched with raspberry pomace was made based on the analysis of characteristic spectral data. Twelve polyphenolic compounds were identified in the bars, the spectral properties of which are presented in Table 1.

UPLC-PDA-MS-MS analysis showed that five polyphenolic compounds were identified in the control bars without additives, four of which were representatives of phenolic acids: caffeic acid glucoside, chlorogenic acid, coumaric acid glucoside, coumaryl-quinic acid (compounds **9**–**12**), and one belonging to the group of flavonols—kaempferol pentoside-rhamnoside (compound **8**). Among the analyzed compounds, chlorogenic acid had the highest concentration (7.48 ± 0.24 mg/L). In the fortified bars, the concentration of these compounds gradually decreased as part of the apple mousse was replaced with raspberry pomace. In the enriched bars, among the phenolic acids, the presence of ellagic acid pentoside (compound **7**) was also found, the concentration of which increased from 1.46 ± 0.02 mg/L in BP5 to 12.73 ± 0.09 mg/L in BP50. In the fortified bars, the two main groups of compounds, characteristic of raspberries, were ellagitannins and anthocyanins. Among the ellagitannins identified were bis-HHDP-glucose, dimer of galloyl-bis-HHDP-glucose (sanguiin H-6), galloyl-bis-HHDP-glucose (compounds **4**–**6**), of which the dominant one was galloyl-bis-HHDP- glucose (from 39.64 ± 0.70 mg/L in BP5 to 286.15 ± 9.47 mg/L in BP50). Among the group of anthocyanin dyes, three cyanidin derivatives (compounds **1**–**3**) were identified, i.e., cyanidin 3-O-glucoside-5-O-glucoside, cyanidin 3-O-glucoside, and cyanidin 3-O-rutinoside. Their concentration depended proportionally on the amount of the additive (Table 1).

### 2.2. Antioxidant Activity

Table 2 shows the antioxidant activity (determined by ABTS^+^, Fe^2+^ chelation, and RP methods) of the bars enriched with raspberry pomace. The ability to neutralize ABTS free radicals increased with the increasing amount of raspberry pomace added to the bars. This tendency occurred in all types of extracts, but the smallest addition of 5% did not cause any statistically significant differences (also in buffer and digest extracts with a 10% addition. The REF values for the ABTS method were very high because the antioxidant properties of digest were significantly higher than those of buffer and ethanolic. This demonstrates the effective release of low molecular weight antioxidants from the food matrix during in vitro digestion. In turn, BAC values were similar for all bar variants and averaged 0.135. Such low values indicate a very high bioaccessibility of compounds with the ability to neutralize free radicals.

The ability to chelate transition metal ions is one of the determinants of antioxidant activity. In this study, the ability of extracts obtained from the bars to chelate iron II ions was determined. Among the tested extracts, the highest activity was determined for digest; however, the chelating power decreased slightly with the increase in the amount of added pomace. In relation to the ethanolic and buffer extracts, a positive relationship was found between the amount of additive and the ability to chelate Fe^2+^ (Table 2). The determined REF coefficients were lower than for ABTS and decreased with the increase in the addition of raspberry pomace. The REF(B/D) coefficients were close to 1, which meant that compounds previously associated with cellular structures may have been released during digestion, but they did not exhibit very strong chelating properties. Changes in pH and enzymatic hydrolysis during in vitro digestion may cause changes in the structure of phenolic compounds, modifying their ability to chelate Fe^2+^. This was also confirmed by the fact that the BAC coefficient values increased from BC to BP50. This may mean that the compounds released after simulated digestion from raspberry pomace do not increase the chelating activity of the active ingredients found in apples. Not all forms of polyphenols bind transition metal ions to the same extent.

The reduction power increased gradually with the increasing share of raspberry pomace in all analyzed extracts, but digest found no statistically significant differences between the fortified bars. The relative extractability coefficients ranged from 1.06 to 1.92, with REF (D/E) decreasing with the increase in the amount of additive, and REF (D/B) was at a similar level for all bar variants. The BAC index was the highest for the control bars (0.83), and the lowest for BP10 and BP20 (0.64) (Table 2).

### 2.3. Potential Inhibitory Activity

All analyzed extracts from the bars showed potential anti-inflammatory properties, manifested by the ability to inhibit the activity of enzymes involved in the metabolism of arachidonic acid, i.e., lipoxygenase and cyclooxygenase.

**Figure 2:**
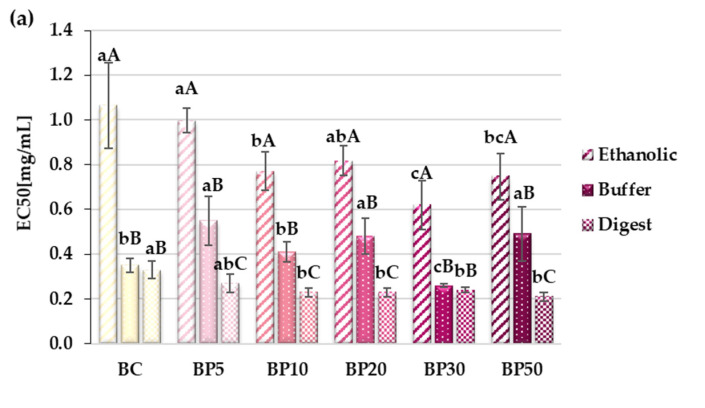

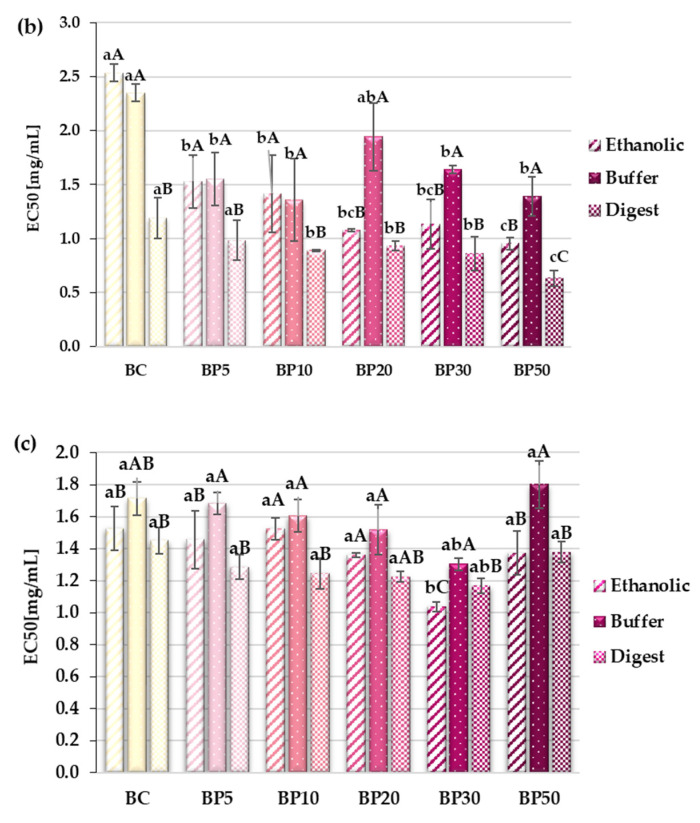
Inhibiting enzyme activity of bars. (**a**) LOXI—lipoxygenase inhibitory activity, (**b**) COXI-2—cyclooxygenase inhibitory activity, (**c**) ACEI—angiotensin converting enzyme inhibitory activity. BC—control bars; BP5–BP50—bars with raspberry pomace addition (from 5 to 50% apple mousse substitution, respectively). All values are mean ± standard deviation for triplicate experiments. Different capital letters indicate statistically significant differences within the same sample but between the different types of extracts (*p* < 0.05). Values denoted by different small letters indicate statistically significant differences between the different samples within the same type of extract (*p* < 0.05).

LOX activity was inhibited least effectively by the ethanolic extracts (the highest EC50 values), and most effectively by the digest extracts. EC50 values for extracts obtained after in vitro digestion were similar for bars fortified in the range from BP10 to BP50 and averaged 0.23 mg/mL (Figure 2a).

Cyclooxygenase-2 generates pro-inflammatory compounds; hence, natural food products with the ability to inhibit its activity are still in demand. In the case of this enzyme, the weakest inhibitory properties were demonstrated by the buffer extracts; however, no linear relationship was found between the EC50 value and the amount of pomace added to the bars. COX-2 activity was inhibited to the greatest extent by extracts after simulated digestion; the lowest EC50 = 0.631 mg/mL was determined for BP50 (Figure 2b). During digestion, phenolic compounds may be released from the food matrix, along with glycosidic bond hydrolysis and the formation of flavonoid and anthocyanin aglycones, whose inhibitory activity towards COX-2 may be stronger. This study also examined the potential antihypertensive properties of apple bars fortified with raspberry pomace. ACE inhibition was quite similar for the ethanolic and digest extracts, but much weaker for the buffer extracts. The BP30 sample showed the strongest inhibitory properties. There was no linear relationship between the increased share of raspberry pomace in the bars and the inhibition of ACE activity. Probably, in the BP50 sample, the extractability of phenolic compounds could have been lower due to the higher share of raspberry seeds. This may have been the reason for the limited release of polyphenols or the saturated ACE-inhibitory effect.

### 2.4. Potential Antiproliferative Activity

**Figure 3:**
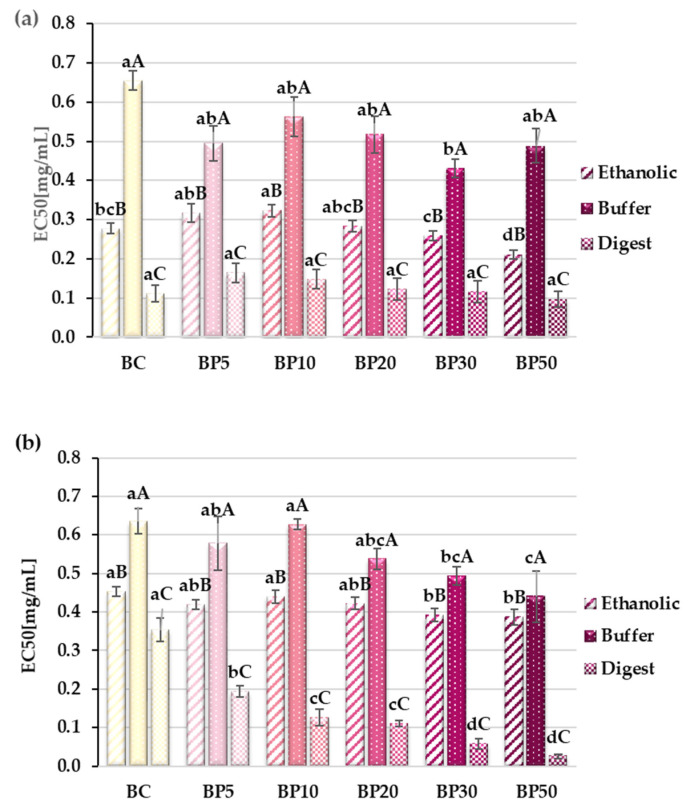
Antiproliferative activity of extracts from bars. (**a**) AGS—gastric adenocarcinoma, (**b**) HT-29—colorectal adenocarcinoma. BC—control bars; BP5–BP50—bars with raspberry pomace addition (from 5 to 50% apple mousse substitution, respectively). All values are mean ± standard deviation for triplicate experiments. Different capital letters indicate statistically significant differences within the same sample but between the different types of extracts (*p* < 0.05). Values denoted by different small letters indicate statistically significant differences between the different samples within the same type of extract (*p* < 0.05).

The graph shows the effect of the prepared extracts from lyophilized bars on the viability of two cancer cell lines: AGS (Figure 3a) and HT-29 (Figure 3b). Biologically active compounds present in the analyzed extracts showed diversified antiproliferative activity. The inhibitory concentration (EC50) values were calculated from semi-logarithmic dose–response curves by linear interpolation. In the case of both lines, the degree of inhibition of cancer cell proliferation depended on the type of extract, in the order digest > ethanolic > buffer, respectively. A linear relationship between the amount of raspberry pomace addition and cell viability was observed only in the case of the extract obtained after simulated digestion in relation to the colon cancer line (HT-29). In the case of the stomach cancer line (AGS), the differences for digest were not statistically significant. For the buffer extracts, the differences between the samples were generally small and there was no obvious relationship between the composition of the bars and the analyzed activity, with the viability of both cell lines most effectively inhibited by samples BP30 and BP50. Analysis of ethanol extracts showed that a 5% addition of pomace was too small to improve or enhance the antiproliferative activity of biologically active compounds from apples. Significant differences in comparison with the control were noted in the case of replacing apple pulp with raspberry pomace in amounts of 30 and 50% (Figure 3a,b). The number of living cells decreased significantly 24 h after treatment of cells with extracts. Apoptosis probably occurred, as the rounding and degradation of cells were visible (Figure S1).

### 2.5. Correlation Analysis

In the case of ABTS radical neutralization capacity, a high positive correlation was observed for all the determined groups of phenolic compounds in all extracts (r > 0.84), except for phenolic acids in the buffer extract, where r = 0.159. This was probably due to the poor extractability of phenolic acids into aqueous solutions. During digestion, phenolic acids can be released from complexes with cell wall elements; hence, the correlation between ABTS and PAC in digest was high (Table 3). Very high correlation coefficients were noted between the content of flavonoids and anthocyanins and the ability to neutralize the ABTS cation radical in all types of extracts. The reduction power was highly positively correlated with the content of anthocyanins and flavonoids only in the ethanolic extract. In the buffer and digest extracts, these coefficients had average values, slightly above 0.6. In turn, the correlation coefficients between the content of flavonoids and anthocyanins and the ability to chelate iron ions were high only in the Buffer extract, while, in digest, a negative one was noted. High and medium correlation coefficients were determined in all extracts between the content of phenolic compounds, flavonoids, phenolic acids, and anthocyanins and the ability to inhibit the activity of enzymes involved in the pathogenesis of inflammation, i.e., LOX and COX-2. Negative values resulted from the inhibitory activity being expressed as an EC50 value, i.e., the lower the value, the higher the ability to inhibit the enzyme. Both anthocyanin and flavonoid content were highly negatively correlated with the ability to inhibit LOX and COX_2 activity in the ethanolic and digest extracts. In turn, the correlation coefficients between ACE inhibition ability were rather low, except for the relationship between the total polyphenol content in the ethanolic extract. In the case of the relationship between the content of individual groups of phenolic compounds and antiproliferative activity, high and very high correlations were noted in the ethanol extracts. In the buffer extracts, the results were similar, except for the correlation calculated for phenolic acids. This confirmed previous observations regarding the solubility of phenolic acids in the buffer. In digest, antiproliferative activity expressed as an EC50 value was also negatively correlated with TPC, FC, PAC, and AC, with higher values calculated for the HT-29 cell line (Table 3).

### 2.6. Consumer Acceptance and Color Analysis

The color analysis in the CIELab system of the prepared bars is presented in Table 4. With the increasing amount of raspberry pomace added to the bars, the L* value (lightness) decreased, while the a* value increased, i.e., the share of red color increased. The values of the b* parameter were positive for all samples, which indicated a greater share of yellow than blue. The highest value was determined for the control bars.

The color saturation of the bars, i.e., the value of the C* parameter considered a quantitative attribute of color, used to determine the degree of difference in shade compared with grey under the same light, was the highest in the BP50 variant. The higher the saturation values, the greater the color intensity of the samples as perceived by humans. The hue angle *h°* parameter decreased with the increasing amount of added pomace, which indicated a change in color towards redder. For comparison, for BC it was 1.22 ± 0.21, and for BP50 0.28 ± 0.016. According to the ΔE evaluation of the fortified bars, the differences were between 21.41 for BP5 and 41.60 for BP50. Color differences between the control and the pomace-enriched bars were noticeable and statistically significant and increased with the increasing pomace addition.

The sensory evaluation carried out by the research team using a five-point scale showed that the addition of a large amount (up to 50%) of raspberry pomace to freeze-dried apple bars had a negative impact on the reception in the consumer group (Table 5). The highest variant (a bar with 50% raspberry pomace) received the lowest scores in the categories of taste (4.26), consistency (4.35), and crispness (4.13), due to its noticeably softer consistency, along with the seeds being noticeable during consumption. The poor taste perception could also have been influenced by the content of raspberry pomace, which caused them to have a sour taste. The highest overall score was given to the BP5 bar variant (with 5% raspberry pomace), whose color was rated the highest (4.91). It also received high scores in the categories of taste (4.70) and consistency (4.78). Analyzing the individual quality features, taste received the highest scores. It was assessed in the range of 4.26–4.78, where the highest score was given to the bar variant with 10% raspberry pomace, and the lowest to the bar variant with 50% raspberry pomace. The consistency of the bars was also rated high. The range was between 4.35 (variant BP50) and 4.83 (variants BP10 and BP20), which proved that the addition of raspberry pomace had no significant impact on the perception of this parameter by the respondents. On the other hand, the aroma was assessed as the worst (range 3.87–4.15), which the consumer group described as “straw”. The average score for all bars was estimated as high. The average scores obtained for all variants were above 4.00, so it can be assumed that the research team found the bars to be sensory appealing. However, the specificity of the raw material used to enrich the bars should be taken into account.

## 3. Discussion

In this paper, an attempt was made to determine the effect of adding raspberry pomace to freeze-dried apple bars on the health-promoting potential of the enriched final product. Attention was also paid to the effectiveness of enriching food products in the context of the potential bioavailability of phenolic compounds. By analyzing the content of total phenolic compounds, it was found that enriching freeze-dried apple bars with raspberry pomace significantly increased the content of these compounds in all the extracts tested (ethanolic, buffer, and digest). The content of phenolic compounds in the control product resulted solely from their presence in the apple pulp. In the studies conducted by Salazar-Orbea et al. (2023) on an industrial scale, the content of total polyphenols in fresh apples was 86.00 ± 9.54 mg/100 g FW, and, in those subjected to gentle short-term heat treatment, it was 106.83 ± 1.30 mg/100 g FW [19]. In turn, TPC of fresh and freeze-dried apple puree from Gold Rush apples was determined at 114 ± 0.7 mg/100 g and 64 ± 1.3 mg/100 g, respectively [20]. These values were slightly lower than in our study, but this depended on the fruit cultivar, degree of ripeness, and extraction method.

The obtained results confirmed the literature data, which indicate the relationship between the share of raspberry pomace in the enriched product and the content of polyphenols present in them. Gluten-free cookie studies conducted by Šarić et al. (2016) showed that replacing the flour mixture with raspberry pomace in the amount of 30% resulted in the recorded total phenolic content of 140.5–145.5 mg/100 g dry matter. The share of raspberry pomace in the product was determined by sensory values, and the additive selected by the authors was the maximum permissible amount, taking into account consumer acceptance and the texture characteristics of the final product [21]. In this study, the content of polyphenols in the enriched products ranged between 139.6 and 483.7 mg/100 g of dry mass in the ethanol extract. After simulated digestion, this amount increased to a maximum of 904.26 mg/100 g, which was almost twice as high as in the case of the other two extracts.

The basis for the production of our bars was apples, which are one of the most commonly consumed fruits in the world. They are easily available, relatively cheap, store well, and can be used in many ways as a fresh snack or after processing. Apples themselves are an excellent source of polyphenols, with various health-promoting properties [22,23]. In turn, raspberries, which are also produced on a large scale, are very delicate fruits, and are difficult to store for a long time; therefore, significant quantities of them are processed into juices and syrups. As a result, pomace (a rich source of polyphenols, including anthocyanins) remains, if left unused is a problem for the environment. The use of waste products (such as fruit pomace), which are a source of bioactive compounds with significant nutritional and pro-health values, as natural additives in the food industry fits perfectly into the idea of sustainable development in food production. The production process of such food should meet three important criteria of being environment- and climate-friendly, economically justified, and socially acceptable. Therefore, their management as an addition to healthy snacks seems to be purposeful [24].

The addition of raspberry pomace significantly enriched the pool and diversity of phenolic compounds. These were polyphenols from the anthocyanin group (three cyanidin derivatives) and ellagitannins. These results corresponded well with data available in the literature. Ripe raspberry fruits, and therefore also their pomace, were dominated by ellagitannins (mainly sanguine H-6) and anthocyanins (most often cyanidin derivatives, less frequently pelargonidins) [25,26,27]. The confirmed strong antioxidant properties of anthocyanins and ellagitannins as anticancer agents additionally justify the choice of raspberry pomace as an additive for the production of freeze-dried bars.

In our studies, we placed particular emphasis on the comparison of extracts before (ethanolic and buffer) and after simulated digestion (digest). The health-promoting activity of phytochemical compounds depends not only on their content in the raw material but also on their chemical structure, interactions with other compounds, and, above all, on their bioavailability, i.e., the possibility of their release from the food matrix in the human digestive tract. The results we obtained indicated that both the concentration of total phenolic compounds, flavonoids, phenolic acids, and antioxidant activity increased after simulated digestion. Only for anthocyanins, the BAC coefficient was above 1, which indicated their low bioaccessibility; however, there was still a visible dependance that the higher the addition of raspberry pomace to the apple base the lower the BAC. The REF factors calculated for the different types of extracts showed that the simulated gastrointestinal tract was more effective in the extraction of phenolics than chemical extraction. This was extremely important, considering the fact that the buffer extraction was not very effective in the extraction of polyphenols. Our results are in agreement with previous studies on bread fortification with plant material [28,29,30], and also particularly with studies on fruit residues [31]. In contrast to our results, is the study by Barros et al. (2020), where, in most cases, the content of polyphenols after digestion was lower than before, i.e., their bioavailability was below 100%. However, it should be taken into account that they digested the extract not the fruit residue itself [32]. As suggested by other authors, bioaccessibility values greater than 100% may be evidence that phenolic compounds are released from the food matrix and/or more complex structures are metabolized to simpler forms [31]. In addition, the pH of the intestinal environment and the action of bile salts can cause changes in phenolic structures, which leads to the formation of new compounds differing from the initial ones in activity and bioavailability [33].

We expected that the fortification of apple bars with raspberry pomace would contribute to the strengthening of the antioxidant potential of the finished product. Most of the tests we conducted confirmed this, although a small (5%) addition of pomace in some cases did not necessarily result in a statistically significant improvement in antioxidant properties. It should be noted, however, that after digestion of the bars, the antioxidant properties were better than before. Simulated digestion of bars fortified with raspberry pomace resulted in a statistically significant increase primarily in the ability to neutralize the ABTS cation radical. Polyphenols from pomace undergo hydrolysis during digestion, which can lead to the formation of compounds with a significantly higher antioxidant potential. Hence, enriching apple pulp with raspberry pomace significantly increased the content of potentially bioavailable antioxidants in the final product. Similar results were obtained in the studies of Andrade et al. [31].

The results of our studies indicated that the bar samples were potential inhibitors of both COX and LOX. It was found that there was a strong correlation between the content of phenolic compounds and the inhibitory activity against LOX and COX.

Natural and safe inhibitors of these enzymes are sought to help reduce the effects of chronic inflammation. Dual COX/LOX inhibitors are believed to be promising novel anti-inflammatory agents [34]. Numerous data from the literature confirm the inhibitory activity of phenolic compounds towards COX-2 [13,34,35,36,37]. Also, much research indicates that phenolic compounds may inhibit both lipoxygenase and cyclooxygenase simultaneously [38,39].

In our study, the greatest COX/LOX inhibitory impact among the tested extracts was on the digested samples. Similar results were obtained in our previous studies [40,41,42]. Probably, the gastrointestinal digestion process increased the release of phenolic compounds with anti-inflammatory activity. These results aligned with findings from other studies that indicated an increase in LOX inhibitory capacities of in vitro digested samples compared with samples before digestion [43].

In our research, by using the UPLC-PDA-MS/MS method, we identified and named 12 polyphenolic compounds contained in pomace bars. The possibility of anti-inflammatory effects by the inhibition mechanism of COX-2 and LOX was predicted for ellagic acid present in fruit pomace-enriched bars. This confirmed molecular docking results obtained by Vyshnevska et al. (2022) [39]. Moreover, dual COX-2/15-LOX inhibitors can play an important role not only in the anti-inflammatory process but also in cancer prevention [44]. COX-2 and 5-LOX are often found to be overexpressed in gastric cancer cells, contributing to the promotion of cancer metastasis and invasion [45,46].

In the treatment of hypertension, ACE inhibition is a crucial therapeutic approach, because ACE facilitates the formation of angiotensin II, a potent vasoconstrictor. In this study, the fortified bars showed generally higher ACE inhibitory activity than the control bars. In a study by Jakubczyk et al. (2021), the hydrolysate obtained from cookies enriched with 1% St. John’s wort following an in vitro digestion process demonstrated increased activity against ACE (EC50 1.24 mg/mL), although the ACE inhibition was not statistically significant [47]. Similarly, this study found that hydrolysate of bars enriched with a 30% raspberry/apple pomace (BP30) had the most significant influence on decreased ACE activity (EC50 1.16 mg/mL), but this effect was also not statistically significant.

ACE inhibition may be related to some specific phenolic compounds included in fruit additives. Contrary to this, Şensu et al. (2021) also did not find any correlation between ACE inhibition activity and phenolic and flavonoid content. It was suggested that ACE inhibition exhibited no correlation with AC and TPC [48]. In our study, with the increase in the amount of fruit pomace additive in the bars, its ability to inhibit ACE increased, but only up to 30% content. In the case of the enrichment with 50% additive, the increase in the inhibiting effect was not observed to the same extent as 30% additive. Therefore, the 30% additive would be sufficient to provide the high inhibition of ACE by our bars. In the study by Erkaya-Kotan (2020), it was observed that the ACE inhibitory activity values obtained for yoghurt with a 1% addition of orange fibers (OF) were higher than those for yoghurts with 1.5% and 2% OF (*p* < 0.05). This was interpreted as a binding interaction between phenolic compounds and milk proteins, which led to a decrease in the bioavailability of polyphenols in yoghurt as ACE inhibitors [49]. In this study, we also observed that the extraction method affected ACE inhibitory activity. Simulated digestion improved the ability of the bars to inhibit ACE. The higher anti-ACE activity of the digested samples could be attributed to the phenolic composition and synergistic effect of several bioactive compounds present in the hydrolysates (proteins, peptides, unsaturated fatty acids) [50].

The studies we conducted showed the antiproliferative potential of the bars against cancer cell lines of the gastrointestinal tract, i.e., stomach cancer line (AGS) and intestinal cancer line (HT-29). Bowen-Forbes et al. (2010) showed that the hexane extract of *R. idaeus* cv. Heritage at a concentration of 250 µg/mL inhibited gastric cancer cells by 22% [51]. Also, D’Errico et al. determined the effects of apple flesh polyphenol extract (AFPE) on cell viability in the AGS (gastric adenocarcinoma) cell line. AFPE inhibited the cell’s viability in a concentration-dependent manner, and the data analysis using semi-logarithmic plots allowed for the determination of IC50 values corresponding to 144 ± 12 μM [52]. Similarly, in the studies of Butkeviciute et al., the extract from whole Kostele apples showed cytotoxicity towards the HT-29 line at the level of EC50 = 113.3 μg/mL [53]. There are also literature reports on the inhibition of intestinal cancer cell proliferation by anthocyanins [54]. Anthocyanin extracts from chokeberry rendered 65% growth inhibition and cell viability of HT-29 cells within 24 h of exposure, suggesting a cytostatic inhibition [55]. Willamette and Meeker raspberry pomace inhibited the proliferation of HT-29 cell lines at EC50 levels of 157 and 180 mg/mL, respectively. The authors suggested that the antiproliferative activity of both investigated pomace extracts could be attributed to the presence of gallic and ellagic acids, quercetin, vitamin C, and their synergistic actions in extract mixtures [56].

Numerous scientific studies indicate the anticancer potential of biologically active compounds, both from whole fruit and from pomace obtained from apples and different berries [55,57,58,59,60]. The mechanisms of this action in relation to gastrointestinal cancers, including stomach and intestine cancer, include the regulation of proliferation, cell cycle, apoptosis, reactive oxygen species (ROS), and anti-inflammatory activities [54].

## 4. Materials and Methods

### 4.1. Chemicals

The following chemical reagents were used for this research: α-amylase, pepsin, pancreatin, bile salts, gallic acid, quercetin, caffeic acid, Folin–Ciocalteau reagent, ABTS (2,2′-azobis(3-ethylbenzothiazoline-6-sulphonate) diammonium salt, ferrozine 3-(2-pyridyl)-5,6-bis-(4-phenyl-sulfonic acid)-1,2,4-triazone), soybean lipoxygenase, linoleic acid, Tween-20, o-phthalaldehyde, potassium ferricyanide K4[Fe(CN)6], formic acid, and acetonitrile HPLC grade. DMEM (Dubelco’s modified Eagle’s medium), FBS (fetal bovine serum), and Pen Strep were purchased from Sigma-Aldrich (Poznań, Poland). COX colorimetric inhibitor screening assay kit was purchased from Cayman Chemical (Ann Arbor, MI, USA). WST 1 cell proliferation reagent was obtained from Abcam (Cambridge, UK). Other analytical grade reagents (iron (II) chloride, TCA (trichloroacetic acid), Na_2_MoO_4_ (sodium molybdate), NaNO_2_ (sodium nitrite), AlCl_3_ × 6H_2_O (aluminum chloride hexahydrate), 98% ethyl alcohol, PBS (buffered saline)) were obtained from Chempur (Piekary Śląskie, Poland). Maltodextrin and citrus/apple amidated pectin were obtained from Agnex (Białystok, Poland).

### 4.2. Research Material

The research material consisted of apple bars enriched with raspberry pomace (*Rubus idaeus* L.). Apples of the “Szampion” variety were purchased from a local market, while raspberries of the “Polana” variety came from a local horticultural farm in the Lublin district.

Raspberry fruits in the amount of 1 kg were mixed for 1 min with a Braun blender (Braun, GmbH, Kronberg im Taunus, Germany), and then placed in a thermal test chamber at 45 °C for 10 min. Then, the obtained raspberry pulp was cooled to 4 °C and centrifuged twice at the same temperature for 15 min at 9000× *g*. The volume of the juice obtained was measured, and the pomace used for the production of bars was weighed and frozen until further processing. Pomace thawed in refrigeration conditions—in the dark at 4 °C—was used for the production of the bars.

The apples were peeled and grated on a vegetable grater immediately before the production of the bars.

### 4.3. Preparation of Freeze-Dried Apple Bars Enriched with Raspberry Pomace

The production of the bars was carried out on a laboratory scale in the following variants:

(1) Bars without the addition of raspberry pomace—BC;
(2) Bars with 5% raspberry pomace—BP5;
(3) Bars with 10% raspberry pomace—BP10;
(4) Bars with 20% raspberry pomace—BP20;
(5) Bars with 30% raspberry pomace—BP30;
(6) Bars with 50% raspberry pomace—BP50.

Different amounts of apple mousse, intended for the individual variants of the bars, were boiled with the addition of 2 g of citric acid (Delecta, Bacalland Sp.z.o.o., Warsaw, Poland), 15 g of maltodextrin, and 6 g of citrus/apple amidated pectin (Agnex, Białystok, Poland). Then, after cooling down, an appropriate amount of raspberry pomace was added (Table 6).

After thorough mixing, 50 g portions were packed into 8.5 cm × 6 cm plastic boxes and frozen at −20 °C, followed by freeze-drying at 8 Pa pressure at −49 °C (FreeZone, Freeze Dry Systems, Labconco, Kansas City, KS, USA).

After receiving the finished product, the bars were hermetically packed and stored in a dry place at room temperature until sensory evaluation and biochemical analyses were carried out. The schematic diagram of the preparation of freeze-dried apple bars enriched with raspberry pomace is included in Supplementary Materials (Figure S2).

### 4.4. Preparation of Extracts

Ethanol extracts were prepared to obtain the maximum amount of phenolic compounds and their identification. However, they did not reflect the physiological state; therefore, extracts in PBS buffer were also prepared to illustrate the state before simulated digestion. The post-digestion extract was intended to clarify the potential bioavailability of polyphenols from freeze-dried apple bars enriched with raspberry pomace.

#### 4.4.1. Preparation of Ethanol Extracts

All variants of the bars were accurately weighed in the amount of 2 g using an AS220/C/2 RADWAG analytical balance (Radwag, Radom, Poland), ground in a mortar, and transferred to 50 mL Falcon tubes. Further steps included the following: three extractions with 15 mL of 50% ethyl alcohol acidified with 0.1% HCl, shaking for 45 min in cooling conditions (4 °C) using a BioSan rotator model Multi Bio RS-24 (Biosan, Riga, Latvia), and centrifugation for 10 min at 9000× *g*. After extraction, the obtained supernatants were collected and made up to a volume of 50 mL with 50% ethyl alcohol acidified with 0.1% HCl. The obtained extracts were stored in a freezer at −60 °C until the determinations were made.

#### 4.4.2. Preparation of PBS Extracts

All variants of the bars were accurately weighed in the amount of 2 g using an AS220/C/2 RADWAG analytical balance (Radwag, Radom, Poland), ground in a mortar, and transferred to 50 mL Falcon tubes. An amount of 15 mL of buffered saline (PBS) was added to each variant of the bar and homogenization was carried out for 2 min, then shaken for 45 min in refrigeration (4 °C) using a BioSan rotator model Multi Bio RS-24 (Biosan, Riga, Latvia), and then centrifuged for 10 min at 9000× *g*. The extraction was performed three times, and the obtained supernatants were collected and made up to 50 mL with buffered saline (PBS).

#### 4.4.3. Preparation of Extracts After Simulated in Vitro Digestion

All the steps in strictly controlled conditions were provided using an Incu-Shaker Mini (Benchmark Scientific, Sayreville, NJ, USA) (temp. 37 °C, no access to light) according to the procedure described by Minekus [61].

The first step was provided to create in vitro conditions for digestion in the oral cavity. The next step was digestion in simulated conditions in the stomach, and the last stage was digestion simulating the conditions in the small intestine (duodenum, jejunum, and ileum). After the last stage of in vitro digestion, all samples were centrifuged at 4 °C at 9000×g, and the obtained supernatants were transferred to 50 mL Falcon tubes (each variant of the bar to the same tube), frozen, and stored for determination.

### 4.5. Phenolics Analysis

#### 4.5.1. Determination of Total Phenolic Compounds (TPCs)

In the determination of total phenolic compounds by the Folin–Ciocalteau method, the phenolic compounds present in the sample were oxidized, while the salts of phosphomolybdic and phosphotungstic acids, which are components of the Folin reagent, were reduced in an alkaline environment. This method used the ability of phenolic compounds to react in color with the Folin reagent, and the resulting product was blue.

The assay was started by adding to 0.1 mL of a suitably diluted 0.4 mL extract diluted with distilled water in a ratio of 1:5 from the Folin–Ciocalteau reagent. After 3 min, 2 mL of 10% Na_2_CO_3_ was added and mixed thoroughly. After 30 min, the absorbance was measured using a UV-VIS spectrophotometer at a wavelength of λ = 725 nm against a standard sample containing ethanol or PBS instead of the extract. The concentrations of total phenolic compounds were read from a standard curve prepared for gallic acid, and the obtained results were converted to gallic acid equivalent (GAE = mg GA/mL) [62].

#### 4.5.2. Determination of Individual Polyphenols from Apple/Raspberry Bars

A Waters ACQUITY UPLC-PDA-MS/MS system (Waters, Milford, MA, USA) with a BEH C18 column (100 mm × 2.1 mm i.d., 1.7 μm, kept at 50 OC, Waters) was used to analyze the polyphenolic compounds. The system consisted of a PDA detector, a tandem quadrupole (TQD) mass spectrometer with electrospray ionization (ESI), and managers of a binary pump, sample, and column. The separation was carried out using the following solvent system: for the anthocyanin—2% formic acid in water (mobile phase A) and 2% formic acid in 40% ACN in water (mobile phase B); for other polyphenolic compounds, a lower concentration of 0.1% formic acid was used. The gradient program was set as follows: 0 min 5% B, from 0 to 8 min linear to 100% B, and from 8 to 9.5 min for washing and back to initial conditions. The sample injection volume and flow rate were 5 μL (partial loop with needle overflow) and 0.35 mL/min, respectively. The TQD parameters were set as follows: capillary voltage 3.5 kV; con voltage 30 V in positive and negative mode, the source was kept at 250 OC and the desolvation temperature was 350 OC; con gas flow 100 L/h; and desolvation gas flow 800 l/h. Argon was used as the collision gas at a flow rate of 0.3 mL/min. The detection and identification of polyphenols were based on specific PDA spectra, mass-to-charge ratio, and fragment ions obtained after collision-induced dissociation (CID). Quantification was determined by external standard calibration. Stock standard solutions of the polyphenols were prepared with methanol. Six calibrators of each standard were prepared by the dilution of stock solutions, and the calibration curve was generated by plotting the peak area ratio of the polyphenol versus the nominal concentration ranging from 0.05 to 5 mg/mL (R^2^ ≤ 0.999).

#### 4.5.3. Determination of the Content of Phenolic Acids (PAC)

The content of phenolic acids was determined using the Arnov method. The following were added directly to each well of a 96-well plate: 192 µL of distilled H_2_O, 32 µL of the tested extract, 32 µL of 0.5% HCl, 32 µL of Arnov’s reagent (10 g Na_2_MoO_4_, 10 g NaNO_2_ in distilled water), and 32 µL of 1 M NaOH, and then the absorbance was measured using the Epoch 2 spectrophotometer by BioTek (Winooski, VT, USA) at the wavelength of λ = 490 nm against the reference sample, which, instead of the tested extract, contained ethanol or PBS. The concentrations of phenolic acids were read from the standard curve prepared for caffeic acid and converted to a caffeic acid equivalent (CAE = µg CA/mL) [63].

#### 4.5.4. Determination of Flavonoid Content (FC)

The content of flavonoids in the tested samples was determined by the Lamaison and Carnat method [64]. The assay involved mixing 150 µL of the extract with 150 µL of 2% AlCl_3_·6H_2_O directly in the wells of a 96-well plate. After 10 min, the absorbance was measured using the Epoch 2 spectrophotometer by BioTek (Winooski, VT, USA) at a wavelength of λ = 430 nm against a standard sample containing ethanol or PBS solution instead of the extract. The assay was performed in three replicates. Flavonoid concentrations were read from the standard curve prepared for quercetin, and the obtained results were converted into a quercetin equivalent (QE = mg Q/mL).

#### 4.5.5. Determination of Anthocyanin Content (AC)

Determination of the anthocyanin content in the analyzed samples was based on the difference in absorbance of the tested solution with different pH. Dilutions of the tested extracts were prepared in KCl buffers at pH = 1 and CH_3_COONa·3H_2_O at pH = 4.5. After 15 min, the absorbance was measured using the Epoch 2 spectrophotometer by BioTek (Winooski, VT, USA) at wavelengths λ = 520 nm and λ = 700 nm against a standard sample containing water instead of the extract. The measurement was performed in three repetitions. Before anthocyanin content measurement, the digest samples were passed through Supelco C-18 cartridges (Sigma-Aldrich, Poznań, Poland) to eliminate the influence of the bile on the quantification.

The absorbance difference was calculated according to the formula [65]:

(1) $$A={\left(A_{520}-A_{700}\right)}_{pH1.0}-{\left(A_{520}-A_{700}\right)}_{pH4.5}$$

The concentration of anthocyanins converted to cyanidin-3-O-glucoside was calculated according to the formula:

(2) $$C(mg\times {mL}^{-1})=\left(A\times MW\times DF\times 1000\right)/(\varepsilon \times 1)$$

where

A—difference in absorbance of the solution at pH 1.0 and pH 4.5;

MW—molecular weight of cyanidin-3-O-glucoside (449.2 g/mol);

DF—sample dilution;

ε—absorption coefficient for cyanidin-3-O-glucoside (26,900 L/cm × mol).

The results obtained were expressed in mg/mL and converted to a cyanidin-3-O-glucoside equivalent.

### 4.6. Antioxidant Activity

#### 4.6.1. Determination of the Ability to Neutralize Free Radicals ABTS^•+^

The spectrophotometric measurement of the ability to neutralize free radicals is based on the ability of antioxidant compounds to neutralize the cation radical generated by ABTS (2,2′-azobis(3-ethylbenzothiazoline-6-sulphonate) diammonium salt) under the influence of sodium persulfate, which is manifested by a decrease in the absorbance of the analyzed solution [66].

The antiradical activity was measured by adding 10 µL of appropriately diluted extract and 270 µL of ABTS directly to the wells of a 96-well plate, followed by a kinetic measurement of absorbance changes over 5 min (measurement every 1 min) using a BioTek Epoch 2 spectrophotometer (Winooski, VT, USA) at a wavelength of λ = 734 nm against a pure solvent standard. The measurement was performed in triplicate [66].

Antiradical activity was calculated according to the formula:

(3) %inhibition=[1−Ap/Ak]×100%

where

%—antiradical activity;

Ap—absorbance of the tested sample;

Ak—absorbance of the control sample.

The results obtained were expressed in µg Trolox/gram dry weight.

#### 4.6.2. Determination of the Ability to Chelate Fe^2+^ Ions

The ability to chelate metal ions (Fe^2+^) was determined spectrophotometrically using a solution of iron (II) chloride and ferrozine as substances with strong binding properties for Fe^2+^ iron ions [67].

The assay consisted of adding 300 µL of appropriately diluted extract and 5 µL of 2 mM FeCl_2_ directly to a 96-well plate, followed by mixing and incubation at room temperature. After 10 min, 10 µL of 5 mM ferrozine was added and incubated again for 10 min. The concentrations of the Fe^2+^/ferrozine complex were determined by measuring the absorbance at a wavelength of 562 nm using the Epoch 2 spectrophotometer by BioTek (Winooski, VT, USA).

The percentage of chelated Fe^2+^ according to the formula:

(4) %inhibition=[1−Ap/Ak]×100%

where

%—ability to chelate Fe^2+^ ions;

Ap—absorption of the test sample;

Ak—absorbance of the control sample (ethanol or PBS solution instead of extract).

The ability to chelate iron ions was read from the standard curve prepared for EDTA and expressed in mg EDTA/g DM.

#### 4.6.3. Determination of the Reduction Power

The method was based on the ability of the analyzed extracts to reduce iron ions Fe^3+^ to Fe^2+^.

Amounts of 0.5 mL of a suitably diluted extract, 0.5 mL of 200 mM sodium phosphate buffer pH 6.6, and 0.5 mL of 1% potassium ferricyanide K_4_[Fe(CN)_6_] were added to a 2 mL Eppendorf tube, after which the samples were mixed and incubated at 50 °C in a thermoblock for 20 min, and then 0.5 mL of 10% trichloroacetic acid (TCA) was added to the samples.

The analysis was performed by adding 125 µL of the obtained supernatant to a 96-well plate, to which 125 µL of distilled water and 25 µL of 0.1% iron (III) chloride were added. The measure of the reduction power was the measurement of absorbance at the wavelength of λ = 700 nm against a control sample containing ethanol or PBS solution instead of the extract. It was performed using the Epoch 2 spectrophotometer by BioTek (Winooski, VT, USA), and the results were read from the standard curve prepared for Trolox (6-hydroxy-2,5,7,8-tetramethylchroman-2-carboxylic acid) and expressed in µM Trolox/g [68].

### 4.7. Theoretical Calculations of Potentially Bioaccessible Phenolic Compounds

The relative extractability factor (REF) was calculated for the content of individual groups of phenolic compounds and their antioxidant activities.

(5) REF=D/E

where D is the concentration of the selected phenolics (TPC, FC, PAC, AC) or antioxidant activity (ABTS, Fe^2+^, RP) in the digest of the bars, E is the concentration of the selected phenolics (TPC, FC, PAC, AC) or antioxidant activity (ABTS, Fe^2+^, RP) in the ethanolic extract of the bars.

(6) REF=D/B

where D is the concentration of the selected phenolics (TPC, FC, PAC, AC) or antioxidant activity (ABTS, Fe^2+^, RP) in the digest of the bars, B is the concentration of the selected phenolics (TPC, FC, PAC, AC) or antioxidant activity (ABTS, Fe^2+^, RP) in the buffer extract of the bars.

The bioaccessibility index (BAC) was calculated to indicate the possibility of antioxidants being released from the food matrix during gastrointestinal digestion.

(7) BAC=B/D

where B is the concentration of the selected phenolics (TPC, FC, PAC, AC) or antioxidant activity (ABTS, Fe^2+^, RP) in the buffer extract of the bars, D is the concentration of the selected phenolics (TPC, FC, PAC, AC) or antioxidant activity (ABTS, Fe^2+^, RP) in the digest of the bars [29,69,70].

### 4.8. Enzyme Inhibition

#### 4.8.1. Potential Anti-Inflammatory Properties

Lipoxygenase (LOXI) inhibitory assay and cyclooxygenase-2 (COXI-2) inhibitory assay was performed by the procedure described earlier [71]. An extract concentration (mg/mL) providing 50% inhibition (EC50) was obtained by plotting the inhibition percentage against the sample concentrations. The ability to inhibit cyclooxygenase 2 (COX-2) was determined with the use of the COX colorimetric inhibitor screening assay kit (Cayman Chemical, Ann Arbor, MI, USA). The results were expressed as EC50—the concentration (mg/mL) that causes 50% inhibition.

#### 4.8.2. Angiotensin-Converting Enzyme Inhibitory (ACEI) Assay

ACE inhibitory activity was measured using the o-phthalaldehyde spectrophotometric assay according to the method of Jakubczyk et al. [72], with some modifications (Karaś et al. [73]). ACE inhibition was expressed in terms of EC50, defined as the sample concentration (mg/mL) required to inhibit 50% of the ACE activity. The EC50 was determined using graphical extrapolation by plotting ACE inhibition as a function of the different sample concentrations.

### 4.9. Antiproliferative Activity

For the antiproliferation assay, 1 mL of freshly prepared extracts was evaporated and concentrated under reduced pressure to dryness (Refrigerated CentriVap Concentrator, Labconco, Kansas City, KS, USA). For the proliferation assay, two cancer cell lines were used: AGS (human Caucasian gastric adenocarcinoma (ECACC No. 89090402) (Sigma-Aldrich, Poznań, Poland)) and HT 29 (human Caucasian colon adenocarcinoma (ATCC HTB-38)) (local distributor of ATCC—(LGC Standards, Łomianki, Poland)). The cells were seeded into a 96-well plate at an initial density of 2 × 10^4^ cells/well and incubated in an air atmosphere humidified with 5% CO_2_ for 24 h at 37 °C. Then, 24 h after seeding, the culture medium (DMEM supplemented with 10% FBS and 1% Pen Strep (Sigma-Aldrich, Poznań, Poland)) was exchanged or replaced with a medium containing ethanolic, buffer, or digest extracts (administered from stock solutions to reach the final concentrations of 1, 0.75, 0.5, and 0.25 mg/mL of culture medium) and the cells were cultured for the next 24 h. After that time, 10 µL of WST 1 cell proliferation reagent (Abcam, Cambridge, UK) was added, and absorbance was measured after a proper time according to the manufacturer’s procedure.

### 4.10. Color Measurement

Color designation was performed using the CIELab method with an EnviSense NH310 colorimeter (EnviSense, Lublin, Poland), equipped with an 8 mm diameter measurement aperture. The instrument was calibrated against a white standard (L* = 95.82, a* = 0.44, b* = 2.5), where L* denoted lightness, a* redness, and b* yellowness. Chroma (*C*), hue angle (*h*°), and total color differences between bars with and without raspberry pomace (Δ*E*) were calculated using the following equations:

*C* = (a*^2^ + b*^2^)^1/2^) (8)

*h*° = arctan (b*/a*) (9)

(10) $$∆E=\sqrt{{\left({∆L}^{*}\right)}^{2}+{\left({∆a}^{*}\right)}^{2}+{\left({∆b}^{*}\right)}^{2}}$$

### 4.11. Consumer Acceptance Test

Sensory analysis was conducted under stable temperature and light conditions by 24 semi-trained panelists selected from workers and students at the University of Life Sciences in Lublin. The features evaluated were shape, color, tenderness, consistency, and smell and taste. The evaluators entered their notes in appropriately prepared tables on the reporting forms. The quality of individual parameters of freeze-dried bars with the addition of raspberry pomace was examined using a five-point hedonistic scale, where note 1 meant the worst and note 5 meant the best quality level. This study conformed to the principles of human protection, human welfare, and ethics. Fresh water was provided for rinsing the mouth.

### 4.12. Statistical Analysis

Statistical analysis was carried out using the Statistica ver. 13.3 software package (StatSoft, Krakow, Poland). The data were shown as mean ± standard deviation. Tukey’s post hoc test, with statistical significance set at *p* ≤ 0.05, was used to compare the mean values of the data obtained from triplicate experiments and measurements (n = 9). The Pearson correlation analysis, performed using Microsoft Excel 2010, was used to indicate a relationship between the content of phenolic compounds and the biological activity of bars enriched with freeze-dried raspberry pomace.

## 5. Conclusions

Results from this study indicate the importance of the apple food matrix, which may also contain bioactive compounds other than phenolics present in the apple extract. Therefore, using apple pulp as a bar base due to its numerous proven health-promoting properties seems purposeful and justified. Moreover, the fortification of apple bars with raspberry pomace positively affects the phenolic content and biological activity of the final product.

Raspberries and their processed products, including pomace, are characterized by a bitter taste, which may reduce the quality of the products to which they are an addition. Therefore, it is recommended to select the appropriate amount of this raw material to obtain a health-promoting effect, but at the same time one that will not reduce the quality of the product. In this case, it can be assumed that the BP30 bar variant best met the assumed criteria, as it received an average score of 4.45 and contained a relatively large amount of raspberry pomace (approx. 15 g/bar).

Hence, such multidirectional actions of phenolic compounds seem to be promising in the prevention of, or even supporting the therapy of, various diseases.

## Figures and Tables

**Table 1:** Polyphenolic identification.

No.	Compound	RT (min)	UV/Vis	[M − H]^+/−^ (*m*/*z*)	MS/MS Fragments (*m/z*)	BC	B5	B10	B20	B30	B50
**1**	Cyanidin 3-O-glucoside-5-O-glucoside	2.69	279, 515	611 ^+^	449, 287	n.d.	1.26 ± 0.01 d	2.36 ± 0.11 d	4.58 ± 0.23 c	8.70 ± 0.61 b	14.61 ± 0.46 a
**2**	Cyanidin 3-O-glucoside	2.93	279, 515	449 ^+^	287	n.d.	1.48 ± 0.01 e	2.73 ± 0.16 d	5.78 ± 0.35 c	9.25 ± 0.92 b	16.44 ± 0.78 a
**3**	Cyanidin 3-O-rutinoside	3.01	275, 507	595 ^+^	287	n.d.	0.49 ± 0.00 c	0.58 ± 0.00 c	0.74 ± 0.03 bc	1.05 ± 0.07 ab	1.47 ± 0.27 a
**4**	bis-HHDP-glucose	3.35	270	270 ^−^	481, 301	n.d.	2.49 ± 0.03 d	4.45 ± 0.21 d	9.15 ± 0.34 c	11.83 ± 0.21 b	17.80 ± 1.44 a
**5**	dimer of galloyl-bis-HHDP-glucose (sanguiin H-6)	3.61	275	275 ^−^	783, 301	n.d.	12.03 ± 0.45 e	23.62 ± 0.28 d	53.30 ± 0.96 c	88.73 ± 2.06 b	135.01 ± 2.32 a
**6**	galloyl-bis-HHDP-glucose	3.86	275	275 ^−^	633, 301	n.d.	39.64 ± 0.70 e	78.70 ± 0.36 d	147.15 ± 2.30 c	190.38 ± 19.17 b	286.15 ± 9.47 a
**7**	Ellagic acid pentoside	4.08	279	279 ^−^	301	n.d.	1.46 ± 0.02 e	2.93 ± 0.12 d	5.23 ± 0.34 c	7.16 ± 0.58 b	12.73 ± 0.09
**8**	Kaempferol pentoside-rhamnoside	2.59	277, 345	563 ^−^	431, 285	0.73 ± 0.02 a	0.49 ± 0.02 a	0.63 ± 0.16 a	0.73 ± 0.05 a	0.63 ± 0.04 a	0.51 ± 0.01 a
**9**	Caffeic acid glucoside	2.91	290	341 ^−^	179	0.37 ± 0.01 b	0.48 ± 0.01 a	0.35 ± 0.04 b	0.34 ± 0.01 b	0.32 ± 0.00 b	0.31 ± 0.03 b
**10**	Chlorogenic acid	3.02	299sh, 326	353 ^−^	191	7.48 ± 0.24 a	6.99 ± 1.08 a	5.95 ± 0.98 ab	4.43 ± 0.33 bc	3.83 ± 0.10 bc	2.75 ± 0.32 c
**11**	Coumaric acid glucoside	3.14	299sh, 316	325 ^−^	163	0.85 ± 0.08 a	0.60 ± 0.01 b	0.60 ± 0.01 b	0.34 ± 0.01 c	0.31 ± 0.02 c	0.27 ± 0.01 c
**12**	Coumarylo-quinic acid	3.74	310	337 ^−^	191	1.47 ± 0.06 a	1.13 ± 0.08 b	0.92 ± 0.01 c	0.83 ± 0.01 c	0.78 ± 0.02 c	0.60 ± 0.01 d
Total						10.89 ± 0.25 f	68.55 ± 0.01 e	123.83 ± 0.69 d	232.61 ± 2.26 c	322.97 ± 19.32 b	488.66 ± 14.49 a

Abbreviations: RT, retention time; [M-H]^+/−^, + and − means ionisation mode—positive (+) and negative (−); m/z, mass-to-charge ratio; UV-Vis, ultraviolet-visible, n.d.—non detected; Data are expressed as mean values ± SD; SD—standard deviation. Mean values within columns with different letters are significantly different (*p* < 0.05) according to the Tukey test.

**Table 2:** Antioxidant properties of apple bars with raspberry pomace.

Antioxidant Activity		Sample					
		BC	BP5	BP10	BP20	BP30	BP50
ABTS^•+^ [mM TE/100 g]	Ethanolic	0.22 ± 0.01 e	0.38 ± 0.06 de	0.45 ± 0.05 cd	0.58 ± 0.05 bc	0.77 ± 0.05 b	1.47 ± 0.14 a
	Buffer	0.39 ± 0.08 c	0.41 ± 0.03 c	0.47 ± 0.13 bc	0.68 ± 0.03 ab	0.85 ± 0.13 a	0.91 ± 0.11 a
	Digest	3.63 ± 0.26 c	3.58 ± 0.38 c	4.65 ± 0.18 b	4.66 ± 0.49 b	4.65 ± 0.22 b	5.58 ± 0.22 a
	REF(D/E)	16.50	9.53	10.31	8.00	6.03	3.80
	REF(D/B)	9.37	8.73	9.81	6.83	5.50	6.14
	BAC (B/D)	0.11	0.11	0.10	0.15	0.18	0.16
Fe^2+^ [mg EDTA/100 g]	Ethanolic	30.84 ± 1.64 ab	29.81 ± 0.68 b	31.42 ± 2.19 ab	28.29 ± 1.80 b	29.13 ± 1.62 b	34.20 ± 0.33 a
	Buffer	36.28 ± 1.29 c	38.35 ± 0.59 bc	40.19 ± 2.16 b	39.72 ± 0.50 b	45.62 ± 0.10 a	46.81 ± 0.07 a
	Digest	47.12 ± 0.16 a	47.10 ± 0.10 ab	46.76 ± 0.30 abc	46.85 ± 0.04 abc	46.69 ± 0.08 cd	46.66 ± 0.05 d
	REF(D/E)	1.53	1.58	1.49	1.66	1.60	1.36
	REF(D/B)	1.30	1.23	1.16	1.18	1.02	1.00
	BAC (B/D)	0.77	0.81	0.86	0.85	0.98	1.00
RP [mM TE/100 g]	Ethanolic	0.95 ± 0.08 e	1.11 ± 0.05 d	1.15 ± 0.01 d	1.52 ± 0.07 c	1.72 ± 0.01 b	2.06 ± 0.02 a
	Buffer	1.19 ± 0.08 b	1.41 ± 0.04 a	1.35 ± 0.02 ab	1.38 ± 0.02 a	1.42 ± 0.03 a	1.42 ± 0.11 a
	Digest	1.83 ± 0.11 b	2.12 ± 0.03 a	2.15 ± 0.04 a	2.17 ± 0.07 a	2.15 ± 0.04 a	2.18 ± 0.09 a
	REF(D/E)	1.92	1.92	1.88	1.43	1.25	1.06
	REF(D/B)	1.54	1.50	1.59	1.57	1.51	1.53
	BAC (B/D)	0.83	0.77	0.64	0.64	0.66	0.66

BC—control (only apple) bars; BP5–BP50—bars with raspberry pomace addition (from 5 to 50% apple mousse substitution, respectively). REF—relative extractability factor, BAC—bioaccessibility index. Means (±SD) followed by different letters are significantly different (n = 9; *p* ≤ 0.05) within the same type of extract.

**Table 3:** Pearson correlation coefficient between polyphenol content values and antioxidant, anti-inflammatory, and antiproliferative activity of different extracts from bars fortified with raspberry pomace.

	Ethanolic			
	TPC	FC	PAC	AC
ABTS^•+^	0.876	0.940	0.984	0.958
Fe^2+^	0.257	0.529	0.486	0.396
RP	0.963	0.929	0.974	0.996
LOXI	−0.808	−0.701	−0.673	−0.779
COXI-2	−0.723	−0.627	−0.793	−0.793
ACEI	−0.783	−0.607	−0.498	−0.605
AGS	−0.818	−0.858	−0.800	−0.821
HT-29	−0.931	−0.902	−0.916	−0.900
	Buffer			
	TPC	FC	PAC	AC
ABTS^•+^	0.968	0.950	0.159	0.985
Fe^2+^	0.988	0.981	−0.052	0.934
RP	0.648	0.614	−0.222	0.724
LOXI	−0.716	−0.746	−0.167	−0.545
COXI-2	−0.501	−0.483	0.703	−0.458
ACEI	−0.200	−0.150	−0.406	−0.385
AGS	−0.725	−0.697	−0.082	−0.799
HT-29	−0.939	−0.934	−0.177	−0.928
	Digest			
	TPC	FC	PAC	AC
ABTS^•+^	0.891	0.846	0.851	0.864
Fe^2+^	−0.887	−0.836	−0.855	−0.882
RP	0.651	0.617	0.611	0.622
LOXI	−0.772	−0.723	−0.732	−0.739
COXI-2	−0.873	−0.841	−0.784	−0.840
ACEI	−0.286	−0.254	−0.365	−0.321
AGS	−0.577	−0.588	−0.639	−0.601
HT-29	−0.886	−0.856	−0.847	−0.869

**Table 4:** Assessment of bars’ color parameters.

Sample	Color Parameters					
	L*	a*	b*	C*	*h°*	Δ*E*
BC	75.88 ± 1.99 a	9.38 ± 0.75 f	25.66 ± 0.40 a	27.37 ± 0.62 d	1.22 ± 0.210 a	
BP5	64.78 ± 1.09 b	23.26 ± 0.62 e	13.79 ± 0.39 b	27.04 ± 0.73 d	0.54 ± 0.003 b	21.41 ± 0.63 e
BP10	58.15 ± 0.84 c	27.94 ± 0.47 d	12.38 ± 0.32 c	30.56 ± 0.40 c	0.42 ± 0.013 c	28.93 ± 0.46 d
BP20	54.77 ± 1.02 cd	30.47 ± 0.65 c	10.68 ± 0.32 d	32.29 ± 0.69 b	0.34 ± 0.006 c	33.42 ± 0.44 c
BP30	51.90 ± 0.53 d	30.87 ± 0.74 b	8.78 ± 0.22 e	32.09 ± 0.77 b	0.28 ± 0.002 e	36.38 ± 0.25 b
BP50	45.49 ± 3.03 e	32.73 ± 0.70 a	9.57 ± 0.61 e	34.10 ± 0.76 a	0.28 ± 0.016 e	41.60 ± 2.50 a

L*—for lightness from black (0) to white (100), a*—from green (−) to red (+), and b*—from blue (−) to yellow (+), C*—chroma*, h°*—hue angle, Δ*E—*total color differences. BC—control bars, BP5–BP50—bars with 5% to 50% raspberry pomace addition, respectively. All values are mean ± standard deviation for triplicate experiments. Means in the rows marked with the same letters do not differ significantly at *p* ≤ 0.05.

**Table 5:** Consumer acceptance of tested bars.

Sample		Quality					
	Shape	Color	Crispness	Consistency	Aroma	Taste	Overall Acceptance
BC	4.06 ± 0.26 a	4.56 ± 0.24 abc	4.43 ± 0.29 ab	4.69 ± 0.08 a	3.94 ± 0.14 bc	4.52 ± 0.20 ab	4.37 ± 0.31 a
BP5	4.26± 0.27 a	4.91 ± 0.26 a	4.41 ± 0.30 ab	4.78 ± 0.09 a	4.02 ± 0.15 abc	4.70 ± 0.20 a	4.51 ± 0.32 a
BP10	4.2 4 ± 0.27 a	3.85 ± 0.20 d	4.63 ± 0.30 a	4.83 ± 0.09 a	4.15 ± 0.15 a	4.78 ± 0.21 a	4.41 ± 0.31 a
BP20	4.17 ± 0.27 a	4.15 ± 0.22 cd	4.57 ± 0.30 a	4.83 ± 0.09 a	4.07 ± 0.15 ab	4.69 ± 0.21 a	4.41 ± 0.31 a
BP30	4.51 ± 0.29 a	4.78 ± 0.25 ab	4.52 ± 0.28 ab	4.54 ± 0.08 a	3.87 ± 0.14 c	4.5 ± 0.21 ab	4.45 ± 0.31 a
BP50	4.18 ± 0.27 a	4.34 ± 0.23 bc	4.13 ± 0.27 b	4.35 ± 0.09 a	4.11 ± 0.14 a	4.26 ± 0.19 b	4.23 ± 0.30 a

BC—control bars, BP5–BP50—bars with 5% to 50% raspberry pomace addition, respectively. All values are mean ± standard deviation for triplicate experiments. Means in the columns marked with the same letters do not differ significantly at *p* ≤ 0.05.

**Table 6:** Variants of freeze-dried apple bars with the addition of raspberry pomace.

**Bar Variants**	**Apple Mousse [g]**	**Raspberry Pomace [g]**	** 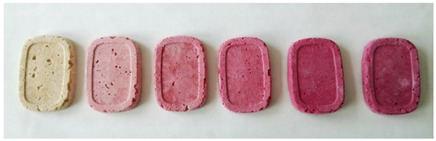 **
BC	200	0	
BP5	190	10	
BP10	180	20	
BP20	160	40	
BP30	140	60	
BP50	100	100	Freeze-dried bars with the addition of raspberry pomace (from the left: BC, BP5, BP10, BP20, BP30, BP50)

## Data Availability

The original contributions presented in this study are included in the article/Supplementary Material. Further inquiries can be directed to the corresponding author.

## Supplementary Materials

The following supporting information can be downloaded at: https://www.mdpi.com/article/10.3390/molecules29235690/s1, Figure S1: Example photos of two cell lines AGS and HT-29 before and after treatment with bar extracts under an inverted microscope. Figure S2: Schematic diagram of the preparation of freeze-dried apple bars enriched with raspberry pomace.
