# Supplementary material for: Raspberry Pomace as a Good Additive to Apple Freeze-Dried Fruit Bars: Biological Properties and Sensory Evaluation
Source: Molecules. 2024 Dec 1;29(23):5690. doi: 10.3390/molecules29235690 (PMC11643381; doi:10.3390/molecules29235690)
Supplement: Supplementary file 1 [file molecules-29-05690-s001.zip › molecules-3318085-supplementary.pdf]

Supplementary material: Raspberry pomace as a good additive to apple freeze-dried fruit bars – biological properties and sensory evaluation

Urszula Szymanowska, Monika Karaś, Anna Jakubczyk, Janusz Kocki, Rafał Szymanowski, Ireneusz Kapusta

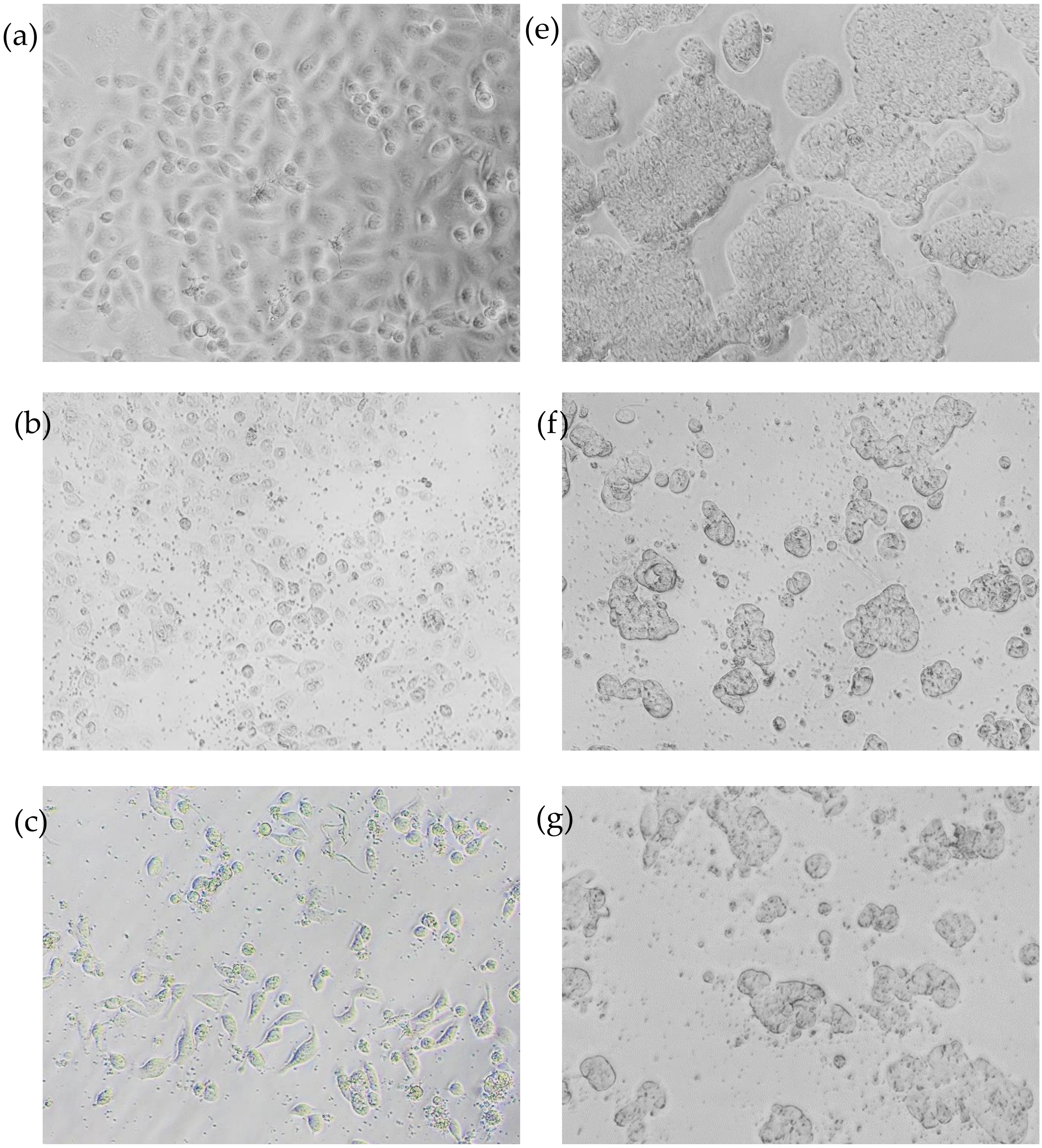

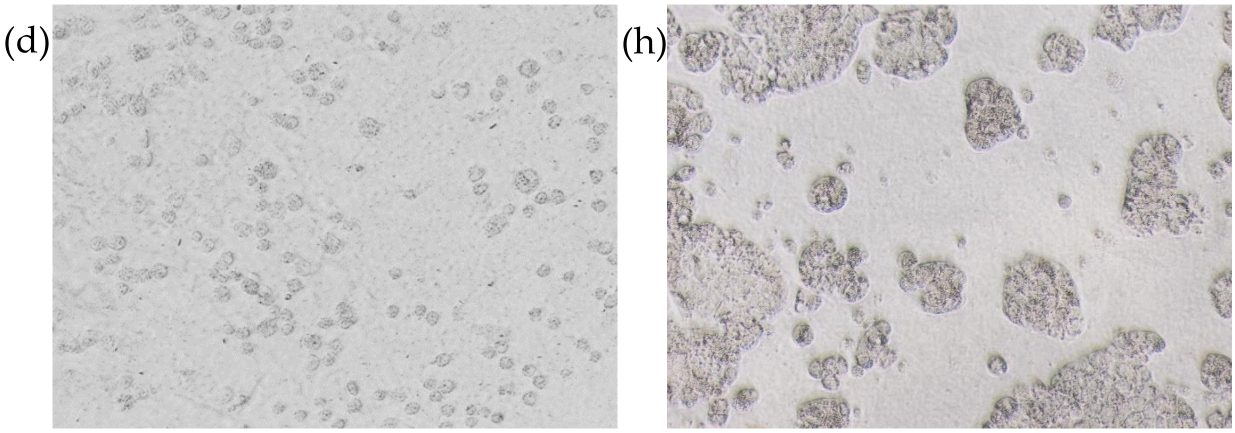

Figure S1. Example photos of two cell lines AGS and HT-29 before and after treatment with bar extracts under an inverted microscope (a): Normal AGS cells. (b): AGS cells 24 hours after treatment with BP30 Ethanolic extract (1 mg/ml) (c): AGS cells 24 hours after treatment with BP50 Ethanolic extract (0.75 mg/ml) (d) AGS cells 24 hours after treatment with BP50 Digest extract (0.25 mg/ml); e): Normal HT-29 cells. (f): HT-29 cells 24 hours after treatment with BP30 Ethanolic extract (0.75 mg/ml) (g): HT-29 cells 24 hours after treatment with BP50 Ethanolic extract (0.75 mg/ml) (h) HT-29 cells 24 hours after treatment with BP50 Digest extract (0.25 mg/ml).

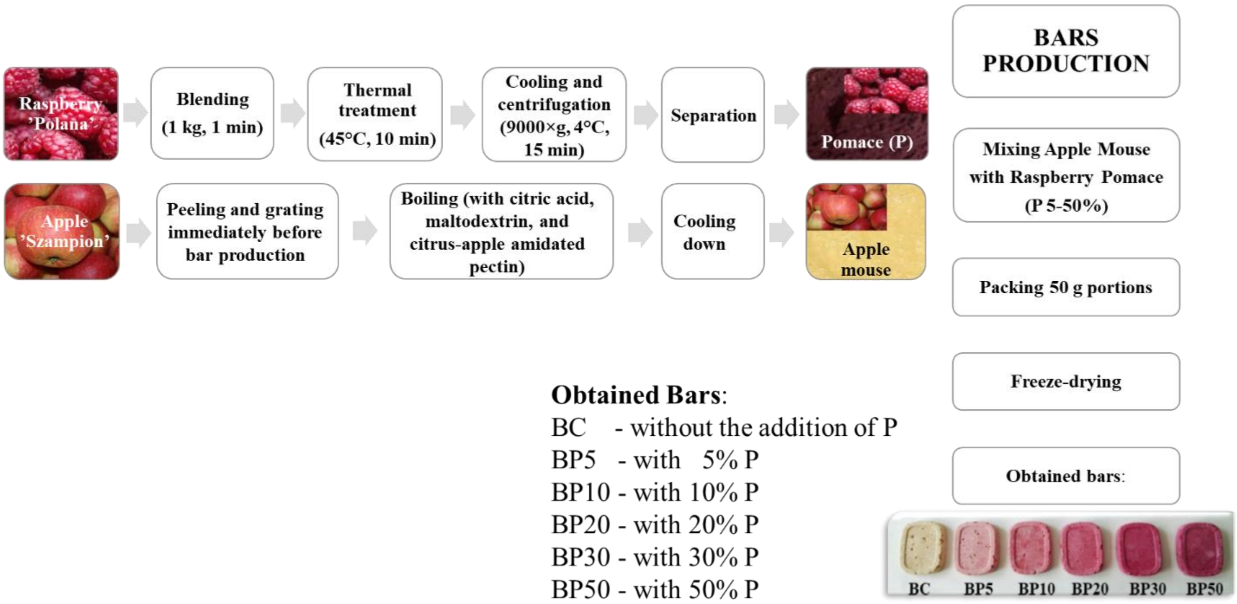

Figure S2. Schematic diagram of the preparation of freeze-dried apple bars enriched with raspberry pomace.
